# Completion of rabies post-exposure prophylaxis in Ouagadougou, Burkina Faso, 2021–2023: A cross-sectional analysis of routine data

**DOI:** 10.1371/journal.pntd.0014437

**Published:** 2026-07-06

**Authors:** Delwendé Samuel Kaboré, Juliette Tranchot-Diallo, Madi Savadogo, Jacques Zoungrana, Nongodo Firmin Kaboré, Anselme Millogo, Abdoul-Salam Ouédraogo, Hervé Hien

**Affiliations:** 1 Université Nazi BONI, Bobo-Dioulasso, Burkina Faso; 2 Centre MURAZ, Institut National de Santé Publique, Bobo Dioulasso, Burkina Faso; 3 Département de Biomed et Santé Publique, Institut de Recherche en Sciences de la Santé (IRSS/CNRST), Ouagadougou, Burkina Faso; 4 Centre Hospitalier Universitaire Sourô SANOU, Bobo-Dioulasso, Burkina Faso; Colorado State University, UNITED STATES OF AMERICA

## Abstract

**Background:**

Rabies remains a major public health concern in low- and middle-income countries, where completion of post-exposure prophylaxis (PEP) is essential to prevent fatal outcomes. Large-scale programmatic evidence from francophone West Africa remains limited.

**Material and Methods:**

We conducted a cross-sectional analytical study using routine data from the Ouagadougou Anti-Rabies Center between 2021 and 2023. Among 8,220 patients receiving rabies prophylaxis, 8,063 (98.1%) received PEP and were included in the analytical component. Completion was defined according to clinical practice, including full vaccination or medically advised discontinuation following risk assessment. Multivariable logistic regression was used to identify factors associated with PEP completion. Dose-specific default patterns were also analyzed.

**Results:**

Overall, 60.2% of patients completed PEP, with a decline from 77% in 2021 to 37% in 2023, coinciding with a documented vaccine stockout. Male sex (aOR=0.89; 95% CI: 0.81–0.98) and age 15–25 years (aOR=0.82; 95% CI: 0.69–0.97) were associated with lower completion. Urban residence was associated with higher completion in the main model (aOR=1.22), although this effect varied significantly by year (interaction p=0.001).

**Conclusion:**

PEP completion in Ouagadougou is primarily driven by socio-demographic and structural factors rather than exposure severity. Early dropout represents a critical intervention point. Strengthening access, decentralizing services, and improving follow-up are essential to enhance completion in endemic settings.

## Author summary

Rabies is a preventable but almost invariably fatal disease once symptoms appear. Post-exposure prophylaxis (PEP), consisting of timely rabies vaccination after a suspected exposure, is highly effective when completed according to recommendations. However, many patients in endemic countries do not complete the full course of treatment, increasing their risk of disease and reducing the effectiveness of rabies control efforts. We analyzed routine data from more than 8,000 patients who received rabies PEP at the Anti-Rabies Treatment Center of Ouagadougou, Burkina Faso, between 2021 and 2023. Overall, six out of ten patients completed treatment, but completion declined sharply over the study period, particularly during a period of vaccine shortage. Men and young adults were less likely to complete treatment than other groups. We also found that many patients discontinued treatment after receiving only the first vaccine dose, highlighting an important point for intervention. Our findings suggest that barriers to PEP completion are driven more by social and health-system factors than by the characteristics of the exposure itself. Improving vaccine availability, decentralizing rabies services, and strengthening patient follow-up could substantially increase treatment completion and contribute to rabies prevention in Burkina Faso and similar settings.

## 1. Introduction

Rabies is a viral zoonosis transmitted to humans through bites, scratches, or contact of saliva from an infected animal with broken skin or mucosa. It remains a major public and animal health concern, affecting more than 150 countries worldwide. Globally, rabies is responsible for an estimated 59,000 human deaths each year, equivalent to approximately one death every 9 minutes, most of which occur in low- and middle-income countries (LMICs) [1]. Once clinical symptoms appear, rabies is almost invariably fatal [2].

Vaccination constitutes the cornerstone of rabies prevention and control in both humans and animals. In humans, prophylaxis involves the administration of multiple doses of rabies vaccine, with or without rabies immunoglobulin, depending on the type and severity of exposure. Post-exposure prophylaxis (PEP) relies on well-established regimens such as the Essen (5-dose intramuscular schedule) or Zagreb (4-dose schedule), while pre-exposure prophylaxis (PrEP) traditionally consists of a 3-dose regimen [3,4]. The effectiveness of these protocols depends critically on timely initiation and full adherence to the prescribed schedule, leveraging the relatively long incubation period of the virus to induce protective immunity [5].

Despite the proven efficacy of rabies prophylaxis, incomplete adherence to PEP remains a major challenge in many endemic settings [6,7]. Several studies conducted in different geographical contexts have documented substantial levels of non-completion and delays in PEP administration. For instance, Arnaud Tarantola and colleagues highlighted significant underestimation of PEP non-completion following dog bites and emphasized the need to better quantify unmet needs in underserved populations [8]. In Rwanda, a recent facility-based study reported suboptimal completion rates and identified socioeconomic and access-related barriers [9]. Similarly, studies from India and China have shown that delayed initiation and incomplete adherence to PEP are influenced by financial constraints, health system limitations, and low risk perception among exposed individuals [5,10]. In Thailand, incomplete or delayed PEP among international travelers has also been documented, underscoring that adherence challenges persist across diverse epidemiological and healthcare contexts [11].

However, beyond these important contributions, several critical gaps remain. First, evidence from francophone West Africa is still scarce, despite the region bearing a significant rabies burden. Most available studies originate from anglophone countries or Asia, limiting the generalizability of findings to francophone health systems with different organizational and operational constraints. Second, many studies rely on relatively small samples or single-facility datasets, whereas large-scale programmatic data analyses remain limited. Third, while delayed initiation of PEP has been relatively well documented, dose-specific default patterns during the vaccination course, which are critical to identify points of dropout, are still insufficiently explored. Finally, few studies have attempted to integrate human prophylaxis data with animal health information within a One Health framework, despite its importance for contextualizing exposure risk and guiding interventions. In Burkina Faso, despite the implementation of standard PEP regimens, systematic monitoring of treatment completion remains limited, and programmatic evidence to guide interventions is scarce.

To address these gaps, the present study leverages large-scale routine data collected at the Ouagadougou Anti-Rabies Center between 2021 and 2023. Specifically, it aims to: (i) assess completion of rabies PEP, (ii) identify dose-specific default patterns along the vaccination schedule, and (iii) analyze factors associated with treatment completion using a multivariable approach. By combining a large programmatic dataset, a focus on operational determinants of adherence, and the integration of available animal surveillance data, this study provides novel, context-specific evidence from francophone West Africa to inform more effective and integrated rabies control strategies.

## 2. Materials and methods

### 2.1. Study design

A cross-sectional analytical study was conducted on patients who visited the rabies treatment center located in Ouagadougou for rabies PEP, while PrEP cases were described but excluded from regression analyses.

### 2.2. Study population and sampling

The study included all patients who received a prescription for rabies prophylaxis at the Ouagadougou Anti-Rabies Center between January 2021 and December 2023, provided that required sociodemographic and clinical information were completely recorded. A total of 8,220 patients were included, comprising 8,063 (98.1%) PEP and 157 (1.9%) PrEP cases.

### 2.3. Data collection

Data were systematically extracted from standardized treatment registries maintained at the Ouagadougou Anti-Rabies Center, covering the period from January 2021 to December 2023. The extracted dataset included key variables such as patient sociodemographic characteristics, details of administered vaccines, and available information regarding the biting animal, allowing identification of patients receiving PEP for analytical purposes.

A trained research assistant performed the initial data collection and electronic entry using KoboCollect (v2024.1.3). To ensure data quality and accuracy, a comprehensive verification protocol was implemented, consisting of: [1] automated validation rules within KoboCollect to prevent entry errors and ensure completeness; [2] systematic review of all electronic entries by the principal investigator against original source documents; and [3] random re-abstraction and verification of 15% of records, confirming a data accuracy rate of 98.7% for core variables.

### 2.4. Study variables

The data collection form consisted of two sections:

- The first section captured sociodemographic characteristics of patients, including sex, age, occupation, and place of residence.
- The second section included variables related to the biting animal and rabies prophylaxis, including type of exposure, vaccination regimen, and follow-up information.

For the purpose of the analytical component, only variables relevant to patients receiving PEP were included in regression analyses.

Two PEP regimens were used at the study site: the Essen protocol (5-dose schedule: days 0, 3, 7, 14, and 28) and the Zagreb protocol (4-dose schedule: 2-1-1). Vaccination could be discontinued after three doses if veterinary observation ruled out rabies risk.

### 2.5. Outcome definition

PEP completion was defined according to WHO recommendations [4], encompassing both full administration of the prescribed vaccination schedule and medically justified discontinuation following veterinary observation. This approach reflects real-world clinical practice and avoids misclassifying patients who followed a valid medical recommendation.

### 2.6. Statistical analysis

Statistical analyses were performed using R software (version 4.2.3). The analytical component was restricted to patients receiving PEP. A multivariable logistic regression model was constructed to identify factors independently associated with completion of the PEP regimen. The outcome variable was binary (completed vs. not completed PEP as defined above).

#### 2.6.1. Variable selection and model building.

Predictor variables were selected a priori based on clinical relevance and existing literature on healthcare access and treatment completion. The initial model included: sex, age category (<5, 5–15, 15–25, ≥ 25 years, with a separate category for ‘not recorded’), area of residence (urban/rural), bite location (head/neck vs. other), and suspected rabies status of the animal (yes/no).

In addition, the variable “year of treatment” (2021, 2022, 2023) was included in the model to account for temporal variations, particularly the documented vaccine stockout in 2023.

All variables were retained in the final model based on an explanatory modeling framework aimed at estimating independent associations rather than prediction, thereby minimizing residual confounding.

#### 2.6.2. Univariable analysis.

Prior to multivariable modeling, univariable logistic regression analyses were performed to assess crude associations between each predictor and PEP completion. Crude Odds Ratios (cOR) with 95% Confidence Intervals and corresponding p-values were reported. These results are presented to provide descriptive insight into the data; however, variable selection for the multivariable model was not based on univariable significance.

#### 2.6.3. Model diagnostics and validation.

The robustness of the multivariable logistic regression model, restricted to patients receiving PEP, was assessed using standard diagnostic procedures. The absence of harmful multicollinearity among predictors was confirmed by calculating Generalized Variance Inflation Factors (GVIF); all adjusted GVIF^(1/(2 × Df)) values were < 1.1, well below the commonly accepted threshold of 2.5.

Although the Hosmer–Lemeshow test suggested a modest lack of fit (p = 0.038), this test is known to be overly sensitive in large samples, and the model was deemed appropriate for association inference.

#### 2.6.4. Handling of missing data.

Cases with missing age information (n = 907, 11.0%) were retained in descriptive analyses but excluded from the multivariable regression model. Given that age is a key sociodemographic confounder and that missingness was unlikely to be completely at random (these patients had markedly lower completion rates), complete-case analysis was preferred to avoid biased estimates from imputation.

#### 2.6.5. Presentation of results.

Results from univariable and multivariable logistic regression analyses are presented as crude Odds Ratios (cOR) and adjusted Odds Ratios (aOR), respectively, with their corresponding 95% Confidence Intervals (CI). Statistical significance was set at a two-sided alpha level of 0.05.

Univariable analyses were conducted for descriptive purposes and to explore crude associations, while all variables included in the multivariable model were selected a priori based on epidemiological relevance and retained regardless of univariable significance to avoid residual confounding.

All analyses adhered to the STROBE guidelines for reporting observational studies.

### 2.7. Ethical considerations

This retrospective analysis of anonymized routine programmatic data was conducted under the official public health authorization of the Municipal Health Department of Ouagadougou (Reference No. 2024–653/CO/M/SG/DGSA/DAGCA/SAA), which oversees the Anti-Rabies Center and permits the use of its data for operational monitoring and research purposes.

The study protocol was formally submitted to the Comité d’Éthique pour la Recherche en Santé (CERS) of Burkina Faso and received a favorable ethical opinion (Deliberation No. **2025-12-590**).

All procedures were conducted in accordance with national ethical guidelines and the principles of the Declaration of Helsinki. Particular attention was paid to the protection of patient confidentiality, and all data were fully anonymized prior to analysis.

## 3. Results

### 3.1. Socio-demographic characteristics of patients

A total of 8,220 patients were included in the study, of whom 48.91% were under 15 years of age. The majority were male (61.06%), corresponding to a male-to-female ratio of 1.56. Most patients resided in urban areas (88.69%). Among rural localities, Saaba and Komsilga recorded the highest numbers of patients (Table 1).

**Table 1:** Sociodemographic characteristics of patients receiving rabies prophylaxis, Ouagadougou, 2021–2023 (N = 8,220).

Characteristic	N	%
**Sex**		
Female	3,201	38.9
Male	5,019	61.1
**Age group (years)**		
<5	1,351	16.4
5–15	2,669	32.5
15–25	958	11.7
≥25	2,335	28.4
Not recorded	907	11.0
**Residence**		
Urban	7,290	88.7
Rural	930	11.3

### 3.2. Geographical distribution of patients under post-exposure prophylaxis

This section presents the spatial distribution of patients who initiated PEP in Ouagadougou between 2021 and 2023. Administrative sectors were categorized according to the number of PEP cases recorded during the study period. This analysis is descriptive and does not imply statistical association or causality.

**Fig 1:**
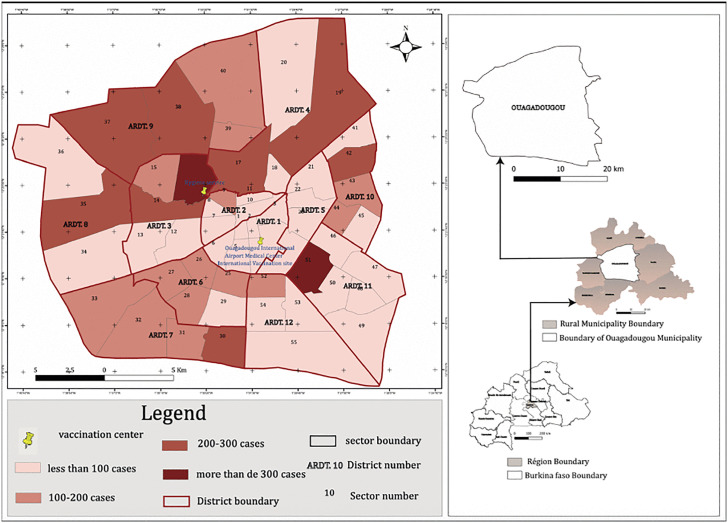
Geographical distribution of patients under post-exposure prophylaxis (2021-2023). Note: Map generated using ArcMap (Esri) and Adobe Illustrator. Administrative boundaries reproduced with permission from the Ouagadougou Municipal Authority (2026).

The map shows that peripheral sectors of Ouagadougou recorded the highest numbers of PEP patients. The two highest concentrations were found in sector 16, which hosts the rabies treatment center, and sector 51. (Fig 1). These patterns should be interpreted cautiously, as they may reflect both true exposure differences and variations in healthcare access or reporting.

### 3.3. Completeness of prophylaxis over time

Overall, 60.2% (4,951/8,220) of patients completed their rabies prophylaxis. A marked decline in completion rates was observed over time, decreasing from 77% in 2021 to 37% in 2023, coinciding with a documented vaccine stockout at the center during part of 2023 (Table 2). When restricted to patients receiving PEP, the completion rate was 60.2% (4,852/8,063). PrEP cases (1.9% of the sample) were described for completeness but excluded from regression analyses.

**Table 2:** Completeness of prophylaxis among patients between 2021 and 2023.

Year	N	Not completed (%)	Completed (%)
2021	2,661	23	77
2022	2,886	34	66
2023	2,673	63	37
**Total**	**8,220**	**39.8**	**60.2**

### 3.4. Characteristics and completion status by prophylaxis type

The characteristics of patients receiving PEP and PrEP, along with their completion status, are presented in Table 3. Among PEP patients (n = 8,063), completion rates varied by bite location and animal rabies status.

**Table 3:** Characteristics and completion status of patients receiving rabies prophylaxis, Ouagadougou, 2021–2023.

Variables	Total n (%)	Completed n (%)	Not completed n (%)
**Post-Exposure Prophylaxis (PEP)**	**8,063 (98.1)**	**4,852 (60.2)**	**3,211 (39.8)**
*Bite location (among PEP)*			
Head/neck	51 (0.6)	36 (70.6)	15 (29.4)
Other	8,012 (99.4)	4,816 (60.1)	3,196 (39.9)
*Animal rabies status (among PEP)*			
Laboratory-confirmed	268 (3.3)	182 (67.9)	86 (32.1)
Suspected/unknown	7,795 (96.7)	4,670 (59.9)	3,125 (40.1)
**Pre-Exposure Prophylaxis (PrEP)**	**157 (1.9)**	**99 (63.1)**	**58 (36.9)**

### 3.5. Univariable analysis of factors associated with PEP completion

Univariable analyses were conducted among patients receiving PEP to explore crude associations between patient characteristics, exposure-related variables, and completion of the vaccination schedule (Table 4).

**Table 4:** Univariable analysis of factors associated with PEP completion, Ouagadougou, 2021–2023.

Variables	Category	Completed n (%)	Not completed n (%)	cOR	95% CI	p-value
**Sex**	Female (Ref)	1987 (62.07)	1214 (37.93)	1.00	–	–
	Male	2964 (59.06)	2055 (40.94)	0.88	0.80–0.96	0.006
**Area of residence**	Rural (Ref)	528 (56.77)	402 (43.23)	1.00	–	–
	Urban	4423 (60.67)	2867 (39.33)	1.17	1.02–1.34	0.023
**Age group (years)**	<5 (Ref)	848 (62.77)	503 (37.23)	1.00	–	–
	5–15	1690 (63.32)	979 (36.68)	1.02	0.89–1.17	0.774
	15–25	558 (58.25)	400 (41.75)	0.83	0.71–0.97	0.021
	≥25	1454 (62.27)	881 (37.73)	0.99	0.87–1.13	0.905
	Not recorded	401 (42.21)	506 (55.79)	0.47	0.40–0.55	<0.001
**Bite location**	Other (Ref)	–	–	1.00	–	–
	Head/neck	36 (70.59)	15 (29.41)	1.60	0.88–2.90	0.120
**Suspected rabid animal**	No (Ref)	1082 (59.98)	722 (40.02)	1.00	–	–
	Yes	3734 (60.15)	2474 (39.85)	1.01	0.91–1.12	0.842

Higher completion rates were observed among patients presenting with head or neck bites and among those exposed to laboratory-confirmed rabid animals. Completion varied across age groups, with lower rates among young adults (15–25 years) and markedly lower completion among patients with missing age data.

Patients residing in rural areas showed slightly lower completion rates compared to urban residents. Differences according to sex were modest.

These unadjusted associations provide descriptive insight into patterns of adherence but were not used to guide variable selection for multivariable analysis.

### 3.6. Interaction analysis: effect modification across subgroups

Interaction analyses were performed using the Breslow–Day test to assess whether associations between selected variables and PEP completion varied across subgroups. As shown in Table 5, no evidence of interaction was observed for most variables. However, a significant interaction was identified between residence and year of treatment (p = 0.001).

**Table 5:** Interaction tests for PEP completion: Breslow–Day test for homogeneity of odds ratios. a. Stratified analysis of the Residence × Year interaction on PEP completion.

Exposure (main factor)	Effect modifier	BD statistic	df	p-value	Conclusion
Sex	Year	0.96	2	0.618	No evidence of interaction
**Residence**	**Year**	**13.98**	**2**	**0.001**	**Evidence of interaction**
Animal rabies status	Year	3.18	2	0.204	No evidence of interaction
Sex	Residence	1.72	1	0.190	No evidence of interaction
Bite location	Year	1.40	1	0.237	No evidence of interaction
**a**					
**Year**	**Rural vs Urban OR**	**95% CI**	**Interpretation**		
2021	0.57	0.45–0.74	Lower odds of completion among rural residents		
2022	0.93	0.71–1.22	No significant difference		
2023	1.00	0.78–1.28	No significant difference		

BD: Breslow–Day test; df: degrees of freedom

To further examine this interaction, a stratified analysis by year was conducted (Table 5a). Rural residence was associated with lower completion in 2021, but no significant differences were observed in 2022 and 2023.

This finding indicates that the rural disadvantage in PEP completion observed in 2021 disappeared in subsequent years, coinciding with the overall decline in completion rates and the vaccine stockout in 2023.

### 3.7. Multivariable analysis of factors associated with PEP completion

In the multivariable analysis, male sex was associated with lower odds of PEP completion (aOR = 0.89, 95% CI: 0.81–0.98; p = 0.018). Individuals aged 15–25 years were also less likely to complete PEP compared with those under 5 years (aOR = 0.82, 95% CI: 0.69–0.97; p = 0.023), while other age groups showed no significant differences. Bite location and animal status were not significantly associated with completion (Table 6).

**Table 6:** Multivariable logistic regression analysis of factors associated with PEP completion, including Year × Residence interaction. a. Stratified effects derived from the interaction model.

Variable	Category	aOR	95% CI	p-value
**Sex**	Male vs Female	0.89	0.81–0.98	0.018
**Age group (years)**	5–15 vs < 5	1.04	0.90–1.19	0.619
	15–25 vs < 5	0.82	0.69–0.97	0.023
	≥25 vs < 5	0.98	0.85–1.12	0.757
**Bite location**	Head/neck vs other	1.33	0.73–2.53	0.363
**Animal status**	Laboratory-confirmed vs other	0.96	0.85–1.08	0.496
**Year × Residence interaction**	2022 × Rural	0.56	0.40–0.79	0.001
	2023 × Rural	0.59	0.42–0.83	0.002
**a**				
**Comparison**	**Year**	**aOR**	**95% CI**	**p-value**
Rural vs Urban	2021	0.57	0.45–0.74	<0.001
	2022	0.93	0.71–1.22	0.603
	2023	1.00	0.78–1.28	0.998

Stratified analyses showed lower completion in rural areas in 2021 (aOR = 0.57; p < 0.001), but no significant differences in 2022 or 2023, suggesting a reduction in rural–urban disparities over time (Table 6a).

A significant interaction between year and residence was observed. Rural residence was associated with lower PEP completion in 2022 (aOR = 0.56; p = 0.001) and 2023 (aOR = 0.59; p = 0.002).

### 3.8. Dose-specific default analysis

Among PEP patients who did not complete prophylaxis **(n = 3,211)**, the majority of defaults **(53.1%, n = 1,704)** occurred after the second dose, followed by **32.4% (n = 1,039)** after the third dose, and **13.5% (n = 433)** after the first dose. An additional **35 patients (1.1%)** who received vaccine vials for administration elsewhere were classified as default based on center records only (Table 7).

**Table 7:** Distribution of defaults by last dose received.

Last Dose Received	Number of Defaults (n)	Proportion of Total Defaults (%)
Dose 1	433	13.5
**Dose 2**	**1,704**	**53.1**
Dose 3	1,039	32.4
Doses taken home*	35	1.1
**Total**	**3,211**	**100.0**

*Note: Patients who received vaccine vials for administration at peripheral health facilities or self-administration. These patients may have completed their schedule elsewhere and were classified as default based on center records only.

## 4. Discussion

This study provides programmatic evidence on adherence to rabies PEP in Ouagadougou between 2021 and 2023, with a particular focus on identifying determinants of treatment completion using routinely collected data. By restricting the analytical component to PEP patients and combining multivariable modeling with dose-specific default analysis, this study offers operationally relevant insights into where and why adherence fails in real-world settings.

### 4.1. Geographical distribution and implications for service delivery

The spatial distribution of PEP cases highlights important inequities in access to care within Ouagadougou. Higher concentrations of patients were observed in peripheral sectors, suggesting both increased exposure risk and differential access to centralized services. Similar observations have been reported in West African settings, where geospatial analyses have helped identify underserved populations and optimize the placement of rabies treatment centers [12].

In this study, areas with frequent human–animal interactions, such as livestock-raising zones, showed higher numbers of PEP administrations, echoing results from Hampson et al. in Tanzania [13]. Conversely, some areas were underrepresented, likely due to underreporting, limited access to health services, or delayed diagnosis [14]. These patterns underline the influence of both true exposure risk and contextual determinants such as geography, infrastructure, and local perceptions.

Addressing these disparities will require decentralizing treatment centers, particularly in peri-urban and rural areas, integrating rabies vaccine into the Expanded Programme on Immunization, and implementing targeted awareness campaigns to improve timely reporting and treatment completion [15]. Enhanced spatial data collection could further refine risk identification and guide resource allocation.

While spatial differences in patient distribution were observed, the absence of formal spatial analysis limits any inference regarding geographic determinants of PEP completion. These findings should therefore be interpreted as indicative patterns rather than evidence of spatial disparities.

### 4.2. PEP completion, temporal trends, and structural barriers

Despite the well-established effectiveness of rabies PEP, approximately 40% of patients initiating treatment in this study did not complete the full vaccination schedule. Given that rabies is almost invariably fatal once symptoms develop, incomplete adherence represents a major public health concern [16,17].

The observed completion rate (60.2%) is broadly consistent with findings from other endemic settings but must be interpreted in light of methodological differences. Estimates derived from routine programmatic data may overestimate completion rates compared with studies using active follow-up. For instance, prospective studies in Senegal and Tanzania reported lower completion rates ranging from 46% to 54%, highlighting potential overestimation in passive surveillance systems [18,19].

Beyond methodological considerations, the consistency of barriers across diverse contexts strengthens the validity of our findings. Financial barriers are a primary constraint, cited by 54.5% of non-compliant patients in Senegal [19]. Vaccine stockouts prevented 15% of exposed persons in Tanzania from initiating PEP [18], while reliance on traditional healers in Nigeria reflects similar structural limitations. These barriers are compounded by transportation difficulties, limited public awareness, and centralized service delivery.

This consistent pattern validates evidence-based strategies that have succeeded elsewhere. Service decentralization addresses geographic access limitations, while intradermal regimens tackle both cost and supply constraints through dose-sparing protocols [20,21]. Integrating these interventions with community-based education and One Health approaches, particularly coordinated dog vaccination, offers a comprehensive framework for addressing the challenges identified in our study. Our analysis suggests that low completion stems from systemic constraints (stockouts, poor follow-up), psychological factors (fear, misinformation), and socio-economic barriers (financial hardship, geographic isolation).

Particularly concerning is the sharp decline in completion from 77% in 2021 to 37% in 2023, driven in part by a major rabies vaccine stockout documented in 2023. Although other contextual factors may have contributed, these were not directly assessed in this study and should therefore be interpreted with caution. Supply issues, vaccine stockouts, or inefficient treatment management could also explain this decline in completion. These findings align with Mbilo et al. [22], who reported persistent PEP access limitations in West and Central Africa, with vaccines largely restricted to urban centers and sold at prohibitive prices (≈USD 15/dose).

Higher adherence rates have been reported in Ghana 95,2% with a retrospective study [23], in India with 73.7% in a longitudinal study of 1,222 patients [24], and more recently by Pal et al., with 73.8% in 122 patients [25], as well as 77.9% in 1,058 patients [26].

These findings highlight the need to implement improvement strategies such as health education programs, improved access to care through vaccine availability, decentralization of rabies treatment centers, and simplification of therapeutic protocols through the implementation of shorter and more cost-effective intradermal vaccination [1,20,21].

### 4.3. Determinants of PEP completion: predominance of structural factors

A key finding is that PEP completion is primarily influenced by socio-demographic and structural factors rather than clinical severity. After adjustment, age 15–25 years (aOR = 0.82) and male sex (aOR = 0.89) were independently associated with lower completion, while bite location and animal rabies status were not significant. This attenuation suggests that while severe exposures motivate treatment initiation, completion depends on overcoming cumulative barriers across multiple visits, a pattern consistent with other LMICs [23,24].

Lower completion among young adults may reflect competing socioeconomic obligations, while the male disadvantage aligns with studies from Senegal and Thailand showing sex differences in healthcare-seeking behavior [11,19].

Our explanatory modeling approach selected variables a priori based on epidemiological relevance. Following identification of a significant Year × Residence interaction, we incorporated interaction terms to avoid model misspecification.

### 4.4. The changing rural–urban gap: a novel finding

A key contribution of this study is the identification of a significant interaction between year of treatment and area of residence (p = 0.001). In 2021, rural residents had significantly lower odds of completing PEP than urban residents (OR = 0.57; 95% CI: 0.45–0.74). However, this rural disadvantage disappeared in 2022 and 2023, with no significant differences observed.

Several hypotheses may explain this pattern. First, the sharp decline in PEP completion from 77% to 37%, driven by a vaccine stockout, may have created a “floor effect” compressing between-group differences. Similar temporary attenuation of disparities during supply disruptions has been documented for yellow fever vaccination in Nigeria [27] and more broadly for routine immunization programs facing stock-outs in African settings [28].

Second, the stockout may have disproportionately affected urban residents accustomed to reliable vaccine availability, while rural residents, already facing persistent shortages, experienced less relative change. This “adaptation to scarcity” phenomenon has been described in health system resilience literature [29].

Third, concurrent COVID-19 pandemic disruptions and the security crisis in Burkina Faso may have differentially affected healthcare-seeking behavior across rural and urban areas [30].

This finding has important methodological implications: failing to test for interactions would have erroneously concluded that residence was not associated with PEP completion (Table 4, p = 0.071), masking the significant rural disadvantage in 2021.

From a programmatic perspective, the disappearance of the rural–urban gap reflects a convergence at unacceptably low completion levels, a “leveling down” rather than genuine equity improvement [31]. True health equity requires improving rural access while maintaining urban outcomes. Strengthening vaccine supply chains, decentralizing PEP delivery, and implementing community-based follow-up are essential strategies.

### 4.5. Dose-specific default analysis

The dose-specific analysis provides important operational insights. More than half of all defaults (53.1%) occurred after the second dose, identifying a critical point of attrition in the vaccination schedule. This finding suggests that while initial uptake of PEP is relatively high, completion rates decline rapidly as indirect costs accumulate and perceived risk diminishes over time.

Such patterns have been documented in other settings and highlight the importance of targeted interventions at specific stages of care [19]. Strategies such as SMS reminders, community health worker follow-up, and financial support mechanisms have shown promise in improving adherence and could be adapted to this context [32].

These findings underscore the importance of a One Health approach to rabies control, in which human PEP completion is closely linked to the performance of animal surveillance and laboratory confirmation systems. Although One Health integration was limited by the structure of routinely collected data, which did not allow formal joint analysis of human and animal datasets, the observed patterns highlight the potential added value of stronger cross-sectoral data integration. The higher completion rates observed following exposure to laboratory-confirmed rabid animals highlight how timely animal diagnosis and information sharing between veterinary and health services can strengthen risk perception and patient compliance. Ultimately, improving canine vaccination coverage and integrating animal and human rabies surveillance are essential to reduce exposure incidence, optimize PEP use, and prevent avoidable human deaths.

### 4.6. Comparison with existing literature

Our finding of a significant Year × Residence interaction is novel in rabies literature. While previous studies consistently reported rural disadvantages in PEP completion [33,34], most employed cross-sectional designs that could not assess temporal effect modification [22].

The convergence of rural-urban rates during the 2023 vaccine stockout, driven by declining urban completion rather than rural improvement, represents a “leveling down” phenomenon, similar to patterns observed during other vaccine supply disruptions. This highlights a critical methodological lesson: failing to test interactions can produce misleading conclusions, as averaging effects across years would have masked the significant rural disadvantage (OR = 0.57) present in 2021 [35].

In West and Central Africa, where recurrent stockouts remain common, our findings underscore the urgency of strengthening supply chains and implementing strategies that maintain equity during disruptions.

### 4.7. Programmatic implications

Priority interventions include: (i) decentralizing PEP delivery to reduce rural–urban disparities [36]; (ii) strengthening patient tracking systems and reminder mechanisms [37]; and (iii) adopting intradermal vaccination regimens, which reduce vaccine volume by 60–80% and visit frequency. In Madagascar, ID delivery reduced vaccine use by approximately 50% compared to intramuscular schedules [38], and simplified two-visit ID regimens directly address the early dropout pattern observed in our study [39].

### 4.8. Study Limitations

Our study has several limitations. First, the single-center design and focus on the capital city limit generalizability to rural areas. Second, we lacked data on socioeconomic status, travel distance, and out-of-pocket costs – key determinants of adherence. Third, the 11% missing age data and the classification of patients who took vials home as ‘default’ may have introduced bias. Furthermore, our definition of PEP completion, which includes medically advised discontinuation following veterinary observation, may have led to a slight overestimation of adherence compared to stricter definitions based solely on the full administration of the vaccination schedule. However, this approach reflects real-world clinical practice and aligns with WHO recommendations, thereby ensuring the operational relevance of our findings. Fourth, the retrospective design prevents causal inference. Finally, we could not assess the proportion of non-completers who actually developed rabies, as no active follow-up was conducted.

## 5. Conclusion

This study highlights persistent challenges to rabies PEP completion in Ouagadougou, with a substantial proportion of patients failing to complete treatment and a marked decline in completion over time.

Contrary to common assumptions, clinical indicators of exposure severity were not independently associated with completion. Instead, socio-demographic and structural factors, particularly age and geographic access, played a more important role in determining completion.

These findings underscore the need to move beyond a purely biomedical approach and address systemic barriers to care. Interventions such as decentralization of services, implementation of reminder systems, and adoption of simplified vaccination regimens should be prioritized.

Strengthening One Health approaches, including improved animal surveillance and mass dog vaccination, will be essential to reduce exposure risk and support progress toward the global goal of eliminating dog-mediated human rabies deaths by 2030 [17].

## Supporting information

S1 File Descriptive analysis of Pre-Exposure Prophylaxis (PrEP) recipients, Ouagadougou, 2021–2023. Definition: PrEP was defined as rabies vaccination administered preventively without documented bite exposure (‘Prévention sans morsure’), including healthcare workers, laboratory staff, veterinarians, and travelers at risk of occupational or travel-related exposure. Content: This file contains two supplementary tables (Tables A and B) presenting descriptive data on PrEP recipients. Table A. Characteristics and completion rates among PrEP recipients (n = 157) – Shows overall completion rates (63.1%), with stratification by year of treatment (2021: 72.3%; 2022: 53.6%; 2023: 65.2%) and by sex (females: 70.3%; males: 56.6%). Table B. Comparison of PEP and PrEP completion rates – Compares PEP (n = 8,063, completion 60.2%) with PrEP (n = 157, completion 63.1%), providing crude odds ratio (OR = 1.13; 95% CI: 0.81–1.57; p = 0.517). Note: PrEP cases were excluded from the main multivariable regression analysis due to their small sample size (1.9% of all patients) and distinct exposure profile (no documented bite). They are presented here for descriptive completeness. Limitation: The small sample size limits statistical power for subgroup analyses within PrEP recipients.
(DOCX)

S2 File Rabies post-exposure prophylaxis (PEP) cases by urban sector, Ouagadougou, 2021–2023. Content: This file contains Table A, which presents the annual and total PEP cases for all 55 urban sectors of Ouagadougou. Table A. Number of rabies PEP cases by urban sector, Ouagadougou, 2021–2023. Sectors are ranked in descending order of total PEP cases over the study period. Burden categories correspond to those used in the spatial distribution map (Fig 1): > 300 cases (very high), 200–300 cases (high), 100–200 cases (moderate), and < 100 cases (low). Annual counts reflect the number of patients initiating PEP at the rabies treatment centre per year. Note: This table includes only patients residing within the 55 urban sectors of Ouagadougou. The remaining 910 PEP cases (11.3% of 8,063 total) were patients from rural areas outside the city and are not assigned to any sector.
(DOCX)

S3 File Data dictionary and README for the anonymized rabies vaccination dataset, Ouagadougou, Burkina Faso, 2021–2023. This file provides documentation for the anonymized dataset used in the study, including variable definitions, coding schemes, descriptions of key study variables, and information on data anonymization procedures. It also describes the study context, ethical approval, and guidance for interpretation and reuse of the dataset.
(DOCX)

S1 Dataset Anonymized dataset of rabies post-exposure prophylaxis (PEP) recipients, Ouagadougou, 2021–20233. This file contains the anonymized individual-level dataset used for the analyses reported in this study.Variables include year of treatment, sex, age group, place of residence (urban/rural), treatment indication, status of the biting animal, number of vaccine doses received, and treatment completion status. Anonymization: All direct identifiers were removed prior to data sharing. No names, addresses, telephone numbers, hospital identifiers, or exact dates are included. Individual ages were recoded into age groups to minimize the risk of indirect participant identification while preserving analytical utility. Data use note: The dataset is provided to sort transparency, reproducibility, and secondary analyses of rabies post-exposure prophylaxis completion in Ouagadougou, Burkina Faso.
(XLSX)
