# Supplementary material for: Completion of rabies post-exposure prophylaxis in Ouagadougou, Burkina Faso, 2021–2023: A cross-sectional analysis of routine data
Source: PLoS Negl Trop Dis. 2026 Jul 6;20(7):e0014437. doi: 10.1371/journal.pntd.0014437 (PMC13362343; doi:10.1371/journal.pntd.0014437)
Supplement: S1 File — Definition: PrEP was defined as rabies vaccination administered preventively without documented bite exposure (‘Prévention sans morsure’), including healthcare workers, laboratory staff, veterinarians, and travelers at risk of occupational or travel-related exposure. Content: This file contains two supplementary tables (Tables A and B) presenting descriptive data on PrEP recipients. Table A. Characteristics and completion rates among PrEP recipients (n = 157) – Shows overall completion rates (63.1%), with stratification by year of treatment (2021: 72.3%; 2022: 53.6%; 2023: 65.2%) and by sex (females: 70.3%; males: 56.6%). Table B. Comparison of PEP and PrEP completion rates – Compares PEP (n = 8,063, completion 60.2%) with PrEP (n = 157, completion 63.1%), providing crude odds ratio (OR = 1.13; 95% CI: 0.81–1.57; p = 0.517). Note: PrEP cases were excluded from the main multivariable regression analysis due to their small sample size (1.9% of all patients) and distinct exposure profile (no documented bite). They are presented here for descriptive completeness. Limitation: The small sample size limits statistical power for subgroup analyses within PrEP recipients. (DOCX) [file pntd.0014437.s001.docx]

**Supporting Information**

**S1 File. Descriptive analysis of Pre-Exposure Prophylaxis (PrEP) recipients, Ouagadougou, 2021–2023**

**Definition:**

PrEP was defined as rabies vaccination administered preventively without documented bite exposure ('Prévention sans morsure'). This category includes healthcare workers, laboratory staff, veterinarians, and travelers at risk of occupational or travel-related exposure.

**Content:**

This file contains two supplementary tables (Tables A and B) presenting descriptive data on PrEP recipients.

| **Table A. Characteristics and completion rates among PrEP recipients (n = 157)** | | | | |
| --- | --- | --- | --- | --- |
|  |  |  |  |  |
| **Variable** | **Category** | **Total (n)** | **Completed n (%)** | **Not completed n (%)** |
| **Overall** | — | 157 | 99 (63.1) | 58 (36.9) |
| **Year** | 2021 | 65 | 47 (72.3) | 18 (27.7) |
|  | 2022 | 69 | 37 (53.6) | 32 (46.4) |
|  | 2023 | 23 | 15 (65.2) | 8 (34.8) |
| **Sex** | Female | 74 | 52 (70.3) | 22 (29.7) |
|  | Male | 83 | 47 (56.6) | 36 (43.4) |

**Table B. Comparison of PEP and PrEP completion rates**

| **Group** | **Total (n)** | **Completed n (%)** | **Crude OR** | **95% CI** | **p-value** |
| --- | --- | --- | --- | --- | --- |
| **PEP** | 8,063 | 4,852 (60.2) | Ref | — | — |
| **PrEP** | 157 | 99 (63.1) | 1.13 | 0.81–1.57 | 0.517 |

**Interpretation:** PrEP recipients showed a slightly higher completion rate (63.1%) compared to PEP recipients (60.2%), but this difference was not statistically significant (OR = 1.13; 95% CI: 0.81–1.57; p = 0.517). Among PrEP recipients, completion varied by year (lowest in 2022: 53.6%) and by sex (females: 70.3% vs males: 56.6%).

**Note:** PrEP cases were excluded from the main multivariable regression analysis due to their small sample size (1.9% of all patients) and distinct exposure profile (no documented bite). They are presented here for descriptive completeness.

**Limitation:** The small sample size limits statistical power for subgroup analyses within PrEP recipients.
