# Supplementary material for: Completion of rabies post-exposure prophylaxis in Ouagadougou, Burkina Faso, 2021–2023: A cross-sectional analysis of routine data
Source: PLoS Negl Trop Dis. 2026 Jul 6;20(7):e0014437. doi: 10.1371/journal.pntd.0014437 (PMC13362343; doi:10.1371/journal.pntd.0014437)
Supplement: S2 File — Content: This file contains Table A, which presents the annual and total PEP cases for all 55 urban sectors of Ouagadougou. Table A. Number of rabies PEP cases by urban sector, Ouagadougou, 2021–2023. Sectors are ranked in descending order of total PEP cases over the study period. Burden categories correspond to those used in the spatial distribution map (Fig 1): > 300 cases (very high), 200–300 cases (high), 100–200 cases (moderate), and < 100 cases (low). Annual counts reflect the number of patients initiating PEP at the rabies treatment centre per year. Note: This table includes only patients residing within the 55 urban sectors of Ouagadougou. The remaining 910 PEP cases (11.3% of 8,063 total) were patients from rural areas outside the city and are not assigned to any sector. (DOCX) [file pntd.0014437.s002.docx]

**S2 File. Rabies post-exposure prophylaxis (PEP) cases by urban sector, Ouagadougou, 2021–2023**

**Content:**

This file contains Table S3, which presents the annual and total PEP cases for all 55 urban sectors of Ouagadougou.

**Note on geographic coverage:**

This table includes only patients residing within the 55 urban sectors of Ouagadougou (7,153 cases). The remaining 910 PEP cases (11.3% of 8,063 total) were patients from rural areas outside the city and are not assigned to any sector.

**Table A.** **Number of rabies PEP cases by urban sector, Ouagadougou, 2021–2023.**

Sectors are ranked in descending order of total PEP cases over the study period. Burden categories correspond to those used in the spatial distribution map (Fig. 1): > 300 cases (very high), 200–300 cases (high), 100–200 cases (moderate), and < 100 cases (low). Annual counts reflect the number of patients initiating PEP at the rabies treatment centre per year.

| **Sector** | **2021** | **2022** | **2023** | **Total (2021–2023)** | **Burden Category** |
| --- | --- | --- | --- | --- | --- |
| Sector 16 | 135 | 146 | 183 | **464** | **> 300** |
| Sector 51 | 138 | 143 | 124 | **405** | **> 300** |
| Sector 42 | 82 | 126 | 99 | **307** | **> 300** |
| Sector 17 | 99 | 117 | 88 | **304** | **> 300** |
| Sector 30 | 89 | 129 | 71 | **289** | **200–300** |
| Sector 19 | 79 | 105 | 79 | **263** | **200–300** |
| Sector 37 | 67 | 106 | 67 | **240** | **200–300** |
| Sector 14 | 81 | 103 | 55 | **239** | **200–300** |
| Sector 38 | 74 | 97 | 67 | **238** | **200–300** |
| Sector 32 | 63 | 98 | 69 | **230** | **200–300** |
| Sector 35 | 65 | 97 | 66 | **228** | **200–300** |
| Sector 26 | 63 | 95 | 54 | **212** | **200–300** |
| Sector 31 | 57 | 90 | 60 | **207** | **200–300** |
| Sector 52 | 62 | 83 | 52 | **197** | **200–300** |
| Sector 15 | 55 | 82 | 59 | **196** | **100–200** |
| Sector 39 | 57 | 77 | 52 | **186** | **100–200** |
| Sector 27 | 56 | 77 | 52 | **185** | **100–200** |
| Sector 44 | 54 | 81 | 45 | **180** | **100–200** |
| Sector 40 | 44 | 61 | 45 | **150** | **100–200** |
| Sector 28 | 40 | 62 | 47 | **149** | **100–200** |
| Sector 43 | 46 | 56 | 40 | **142** | **100–200** |
| Sector 33 | 40 | 60 | 41 | **141** | **100–200** |
| Sector 25 | 37 | 51 | 46 | **134** | **100–200** |
| Sector 9 | 33 | 49 | 41 | **123** | **100–200** |
| Sector 5 | 26 | 33 | 31 | **90** | **< 100** |
| Sector 20 | 25 | 33 | 32 | **90** | **< 100** |
| Sector 41 | 23 | 34 | 31 | **88** | **< 100** |
| Sector 24 | 24 | 30 | 30 | **84** | **< 100** |
| Sector 29 | 22 | 30 | 27 | **79** | **< 100** |
| Sector 36 | 21 | 30 | 27 | **78** | **< 100** |
| Sector 13 | 21 | 30 | 26 | **77** | **< 100** |
| Sector 7 | 22 | 27 | 27 | **76** | **< 100** |
| Sector 49 | 22 | 28 | 25 | **75** | **< 100** |
| Sector 12 | 20 | 27 | 25 | **72** | **< 100** |
| Sector 6 | 19 | 25 | 25 | **69** | **< 100** |
| Sector 23 | 19 | 25 | 25 | **69** | **< 100** |
| Sector 21 | 19 | 24 | 25 | **68** | **< 100** |
| Sector 45 | 19 | 23 | 24 | **66** | **< 100** |
| Sector 34 | 18 | 22 | 23 | **63** | **< 100** |
| Sector 18 | 17 | 22 | 24 | **63** | **< 100** |
| Sector 8 | 17 | 22 | 23 | **62** | **< 100** |
| Sector 47 | 17 | 21 | 23 | **61** | **< 100** |
| Sector 11 | 16 | 20 | 22 | **58** | **< 100** |
| Sector 46 | 15 | 18 | 19 | **52** | **< 100** |
| Sector 53 | 14 | 17 | 20 | **51** | **< 100** |
| Sector 10 | 9 | 19 | 10 | **38** | **< 100** |
| Sector 48 | 18 | 5 | 11 | **34** | **< 100** |
| Sector 4 | 12 | 9 | 12 | **33** | **< 100** |
| Sector 22 | 12 | 11 | 10 | **33** | **< 100** |
| Sector 1 | 8 | 10 | 8 | **26** | **< 100** |
| Sector 55 | 7 | 6 | 11 | **24** | **< 100** |
| Sector 3 | 7 | 4 | 10 | **21** | **< 100** |
| Sector 2 | 7 | 5 | 5 | **17** | **< 100** |
| Sector 54 | 5 | 3 | 6 | **14** | **< 100** |
| Sector 50 | 5 | 4 | 4 | **13** | **< 100** |
| **Total** | **2122** | **2808** | **2223** | **7153** | **55 sectors** |
