# Supplementary material for: Completion of rabies post-exposure prophylaxis in Ouagadougou, Burkina Faso, 2021–2023: A cross-sectional analysis of routine data
Source: PLoS Negl Trop Dis. 2026 Jul 6;20(7):e0014437. doi: 10.1371/journal.pntd.0014437 (PMC13362343; doi:10.1371/journal.pntd.0014437)
Supplement: S3 File — This file provides documentation for the anonymized dataset used in the study, including variable definitions, coding schemes, descriptions of key study variables, and information on data anonymization procedures. It also describes the study context, ethical approval, and guidance for interpretation and reuse of the dataset. (DOCX) [file pntd.0014437.s003.docx]

**S3 File. Data Dictionary and README for the anonymized rabies vaccination dataset, Ouagadougou, Burkina Faso, 2021–2023**

Description

This file provides documentation for the anonymized dataset accompanying the article "Completion of rabies post-exposure prophylaxis in Ouagadougou, Burkina Faso, 2021–2023: a cross-sectional analysis of routine data".

The dataset contains anonymized individual-level records extracted from routine rabies vaccination registers maintained at the Rabies Treatment Centre of Ouagadougou between January 2021 and December 2023.

Data anonymization

To protect participant confidentiality, all direct identifiers were removed before data sharing. The dataset does not contain names, addresses, telephone numbers, hospital identifiers, national identification numbers, or exact dates. Individual ages were recoded into age groups to reduce the risk of indirect participant identification while preserving analytical utility.

Variables included in the dataset

| **Variable** | **Description** |
| --- | --- |
| year | Year of treatment initiation (2021–2023) |
| sex | Sex of patient (Male/Female) |
| age_group | Age category (<5, 5–14, 15–29, 30–44, ≥45 years) |
| residence | Place of residence (Urban/Rural) |
| treatment_type | Type of rabies prophylaxis received (PEP or PrEP) |
| animal_status | Status or outcome of the biting animal as recorded in routine registers |
| doses_received | Number of rabies vaccine doses received |
| completion_status | Treatment completion status (Completed/Not completed) |

Definitions

Post-exposure prophylaxis (PEP): Rabies vaccination administered following a documented animal bite or other exposure potentially associated with rabies transmission.

Pre-exposure prophylaxis (PrEP): Rabies vaccination administered preventively without documented bite exposure, including vaccination of healthcare workers, laboratory personnel, veterinarians, and travellers at increased risk of rabies exposure.

Treatment completion: Completion of the rabies vaccination schedule according to national recommendations in force during the study period.

Ethics

The study protocol was reviewed and approved by the Comité d’Éthique pour la Recherche en Santé (CERS) of Burkina Faso (Deliberation No. 2025-12-590). Data were analyzed and shared in anonymized form.

Purpose of data sharing

The dataset is provided to enhance transparency, reproducibility, and secondary analyses related to rabies post-exposure prophylaxis completion in Burkina Faso.

Citation

Users of this dataset should cite both the associated article and the supplementary dataset when using these data in future research.
